# Neurofilament light chain and retinal layers' determinants and association: A population‐based study

**DOI:** 10.1002/acn3.51522

**Published:** 2022-03-04

**Authors:** Davide Garzone, Robert P. Finger, Matthias M. Mauschitz, Marina L. S. Santos, Monique M. B. Breteler, N. Ahmad Aziz

**Affiliations:** ^1^ Population Health Sciences German Center for Neurodegenerative Diseases (DZNE) Bonn Germany; ^2^ Department of Ophthalmology, Faculty of Medicine University of Bonn Bonn Germany; ^3^ Institute for Medical Biometry, Informatics and Epidemiology (IMBIE), Faculty of Medicine University of Bonn Bonn Germany; ^4^ Department of Neurology, Faculty of Medicine University of Bonn Bonn Germany

## Abstract

Both retinal atrophy measured through optical coherence tomography and plasma neurofilament light chain (NfL) levels are markers of neurodegeneration, but their relationship is unknown. Therefore, we assessed their determinants and association in 4369 participants of a population‐based study. Both plasma NfL levels and inner retinal atrophy increased exponentially with age. In the presence of risk factors for neurodegeneration (including age, smoking, and a history of neurological disorders), plasma NfL levels were associated with inner retinal atrophy and outer retinal thickening. Our findings indicate that inner retinal atrophy can reflect neuroaxonal damage as mirrored by rising plasma NfL levels.

## Introduction

Both plasma neurofilament light chain (NfL) levels and retinal layers' measures assessed through optical coherence tomography (OCT) have emerged as sensitive markers of neurodegeneration.^1^, ^2^ NfL is a blood‐based biomarker, released in the circulation upon neuroaxonal damage, whereas the retina is an extension of the brain susceptible to similar injuries. Both markers change with advancing age, likely reflecting age‐related neuronal loss,^3^, ^4^ and are considered sensitive but unspecific markers in several neurological diseases.^5^, ^6^, ^7^ In the general population, baseline blood NfL levels were recently shown to predict future cognitive decline and brain atrophy.^3^ Measures of inner retinal layers, the main location of retinal neurons, including the ganglion cell layer (GCL) and inner plexiform layer (IPL), are associated with brain volume.^7^, ^8^ Although a recent study investigated the association between plasma NfL levels and retinal measures in a cohort of multiple sclerosis patients,^9^ normative population‐based data regarding this relationship are still lacking. Therefore, using a population‐based design, we aimed to: First, compare the determinants of plasma NfL levels and volume of GCL, the retinal layer most closely reflecting brain volume^8^; and second, examine the association between different retinal layers' measures and NfL levels, and assess whether their relation is modified by risk factors for neurodegeneration.

## Methods

### Study population

We analyzed baseline data from the first 5000 participants of the Rhineland Study, a population‐based cohort study in Bonn, Germany.^4^ Only the right eye was included per participant. We excluded participants with missing or low‐quality NfL (N = 397) and/or right eye OCT data (N = 200). Furthermore, we excluded values larger than 3 standard deviations from both the mean of log‐transformed NfL levels (N = 27) and retinal volume (N = 28), leaving a sample of 4369 subjects for data analysis.

### Refraction and retinal layers

We assessed retinal layers using Spectralis spectral domain‐OCT (Heidelberg Engineering, Heidelberg, Germany). Our workflow and OCT segmentation algorithm have been detailed before.^4^ In brief, volumes of six macular layers, total retinal volume, and peripapillary retinal nerve fiber layer (pRNFL) thickness were computed using the inbuilt segmentation algorithm of the Heidelberg Eye Explorer (HEYEX).

### Neurofilament light chain

Blood samples were collected in Vacutainer EDTA tubes and centrifuged at 2000 *g* for 10 minutes at room temperature and plasma samples were aliquoted and stored at −80 °C. NfL levels were assessed using the Simoa^®^ NF‐light Kit (103186) in a HD‐1 Analyzer (Quanterix, Billerica, USA), following manufacturer's instructions.^10^

### Other covariates

Blood volume was calculated with Nadler's formula.^11^ Glomerular filtration rate (GFR) was estimated based on the CKD‐EPI formula.^12^

### Statistical analysis

Data are summarized as mean ± standard deviation (SD) or counts with proportions, for continuous and categorical variables, respectively. We adjusted NfL levels for batch and lot using a generalized mixed‐effects model and used the log‐transformed residuals in subsequent analyses. First, we assessed the effects of age, age‐squared, sex, GFR, smoking status, blood volume, systolic blood pressure (SBP), history of ocular diseases and neurological disorders on both GCL volume and plasma NfL levels. Then, we investigated the relationship between retinal measures (independent variables), and plasma NfL levels (outcome) using three different models: Model 1 was based on univariate regression estimates, Model 2 was adjusted for GFR, blood volume, spherical equivalent, ocular diseases and sex, Model 3 was additionally adjusted for age and age‐squared. Lastly, we assessed effect modification through the inclusion of an interaction term in the fully adjusted models. All statistical analyses were performed in R (base version 1.4.1106) and *p*‐values <0.05 were considered statistically significant after false discovery rate adjustment for multiple comparisons.^13^

## Results

### Demographics

The mean (SD) age of the study population was 55.1 years (±13.8, range: 30.2–94.1) and 2450 participants (56%) were female. The mean (SD) GFR, SBP, and blood volume were 90.8 (18.7), 126.1 (15.9) and 4.7 (0.9), respectively; 531 (12.1%) participants were current smokers. The mean retinal volume was 8.6 mm^3^ (0.4) and the median NfL value was 7.65 pg/ml (interquartile range: 5.6–11.0). Participants with missing data were significantly older, showed thinner retinas and higher NfL levels. A history of ocular disorders was reported in 99 participants (glaucoma (*N* = 91) and diabetic retinopathy (*N* = 8)). Intermediate age‐related macular degeneration (iAMD) graded on fundoscopy was observed in 135 individuals, while no individuals with late AMD were included in the study population. Neurological disorders were reported by 113 participants (stroke (*N* = 67), dementia (*N* = 5), multiple sclerosis (*N* = 23), and Parkinson's disease (*N* = 18)).

### Determinants of plasma NfL and retinal volume

**Figure 1:**
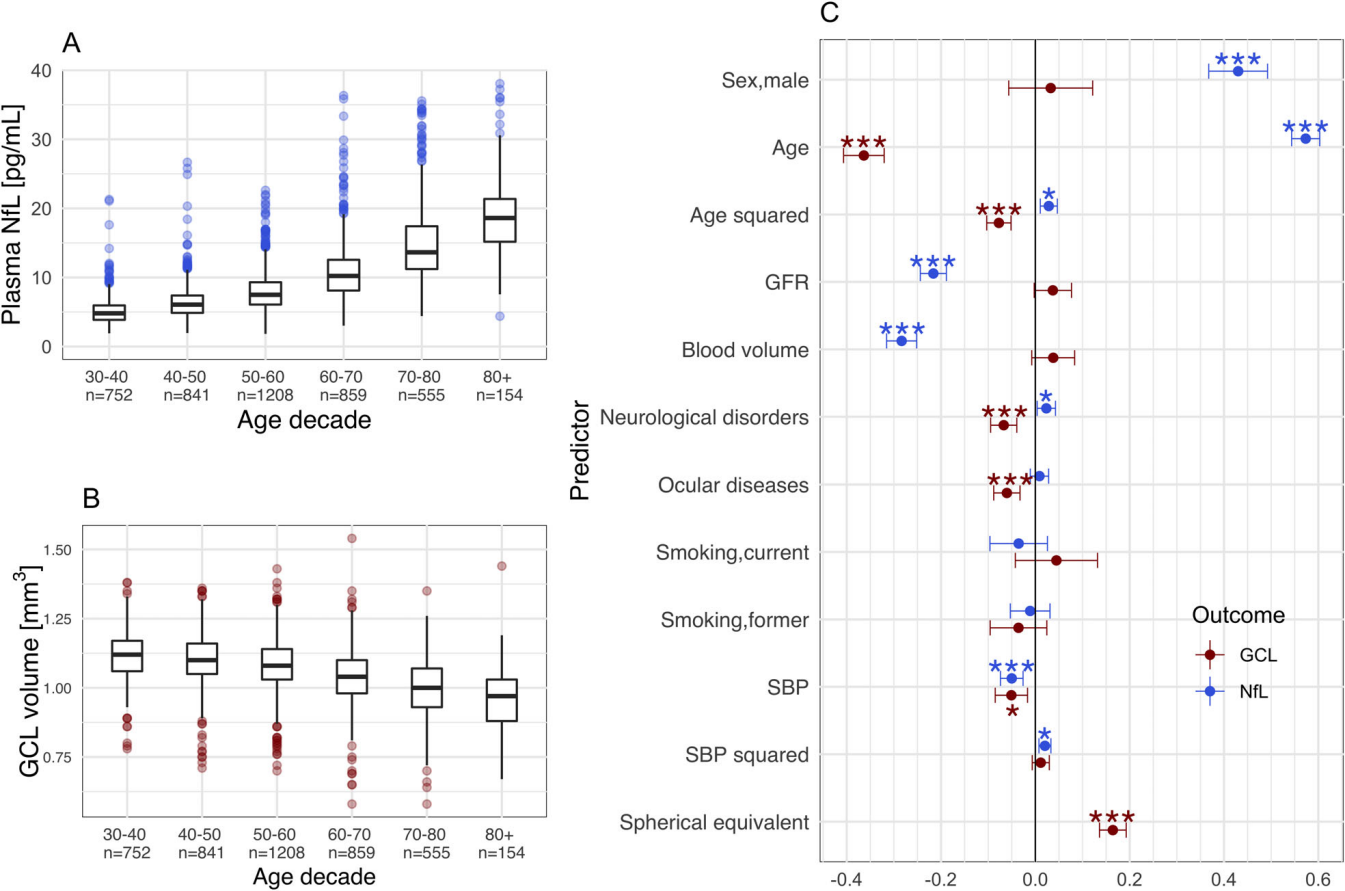
Differential age‐effects and determinants of plasma NfL levels and GCL volume**.** Advancing age was associated with an exponential increase in plasma NfL levels (A), and an exponential decrease in GCL volume (B). Standardized estimates derived from a multivariable regression model, assessing the independent effect of each specified determinant, showed both similarities and differences between determinants of plasma NfL levels and GCL (C). Abbreviations: GFR, glomerular filtration rate; SBP, systolic blood pressure; BMI, body‐mass index; GCL, ganglion cell layer volume; NfL, neurofilament light chain. * q < 0.05, ** q < 0.001, *** q < 0.0001. [Colour figure can be viewed at wileyonlinelibrary.com]

We found that advancing age was the strongest determinant of both measures (1 SD increase in age was associated with 0.57 SD increase in plasma NfL levels (95% CI = [0.54–0.60], *p*‐value = <0.0001) and 0.36 SD decrease in GCL volume (95% CI = [−0.40 to −0.32], *p*‐value = <0.0001) (Figure 1). Plasma NfL increased by 3.0%/year and levels in the eldest group were 3.8 times higher compared to those in the youngest group (Figure 1A), while mean GCL volume decreased by 0.30% per year and showed a decline of 13.9% from the youngest to the eldest decade (Figure 1B). The highest relative change per decade for both biomarkers occurred in the seventh decade: Plasma NfL increased by 35.2% and GCL volume decreased by 3.74%. A history of neurological disorders was significantly associated with both biomarkers (Figure 1C). GFR and blood volume were important determinants of NfL (std. effect size [95% CI] for GFR = −0.216 [−0.244 to −0.189], for blood volume = −0.283 [−0.315 to −0.252]), while SBP had a quadratic relationship with plasma NfL but a linear one with GCL volume. No effect of smoking was noted. History of ocular diseases and refraction with a lower spherical equivalent were associated with thinner GCL (Figure 1C).

### The relation between plasma NfL and retinal measures

All inner neuronal retinal layers (including GCL and IPL) and total retinal volume were negatively associated with plasma NfL levels in the univariate and partially adjusted models; however, the direction of these associations reversed after adjustment for age (Table 1). In contrast, retinal pigmented epithelium volume (RPE) remained positively associated with NfL levels even after age adjustment (Table 1).

**Table 1:** Association between total retinal volume and individual retinal layers with plasma NfL.

Method	Model 1, unadjusted^†^	Model 2, partially adjusted^†^	Model 3, fully adjusted^†^	Interaction terms			
Predictor	Std. Beta (95% CI)			Age	Neurological disorders	Smoking	Systolic blood pressure
RPE	0.027 (−0.003 to 0.057)	0.049*** (0.026 to 0.071)	0.028* (0.008 to 0.047)	0.006 (−0.013 to 0.024)	−0.010 (−0.028 to 0.008)	0.026 (−0.033 to 0.086)	0.016 (−0.003 to 0.035)
IPL	−0.259*** (−0.288 to −0.231)	−0.079*** (−0.102 to −0.056)	0.024* (0.003 to 0.044)	0.006 (−0.013 to 0.026)	−0.017**‘** (−0.033 to −0.000)	−0.078* (−0.138 to −0.018)	−0.008 (−0.027 to 0.011)
GCL	−0.297*** (−0.326 to −0.269)	−0.098*** (−0.122 to −0.074)	0.024* (0.002 to 0.045)	0.005 (−0.015 to 0.025)	−0.020* (−0.036 to −0.003)	−0.078* (−0.138 to −0.017)	−0.011 (−0.030 to 0.008)
pRNFL	−0.166*** (−0.196 to −0.137)	−0.044** (−0.067 to −0.021)	0.019 (−0.001 to 0.039)				
TRET	−0.211*** (−0.240 to −0.182)	−0.056*** (−0.079 to −0.033)	0.021 (0.001 to 0.041)				
ONL	−0.116*** (−0.146 to −0.087)	−0.044** (−0.067 to −0.021)	0.007 (−0.013 to 0.027)				
OPL	0.026 (−0.004 to 0.056)	0.015 (−0.008 to 0.037)	−0.003 (−0.023 to 0.016)				
INL	−0.171*** (−0.200 to −0.142)	−0.071*** (−0.094 to −0.048)	−0.003 (−0.023 to 0.018)				

Abbreviations: GCL, ganglion cell layer volume; INL, inner nuclear layer volume; IPL, inner plexiform layer volume; ONL, outer nuclear layer volume; OPL, outer plexiform layer volume; pRNFL, peripapillary retinal nerve fiber layer thickness; RPE, retinal pigmented epithelium volume; TRET, total retinal volume.

* q < 0.05

** q < 0.001

*** q < 0.0001 after multiple comparison correction.

^†^ Model 1: unadjusted univariate regression analysis, Model 2: adjusted for GFR, blood volume, spherical equivalent, ocular diseases, sex, Model 3: additional adjustment for age and age‐squared. In Model 3, we also checked for multi‐collinearity by examining the variance inflation factors, which did not indicate any relevant degree of collinearity; all variance inflation factors were <<5: GCL = 1.26; sex = 2.6; SE_OD = 1.1; ocular diseases = 1.0; GFR = 2.1; blood volume = 2.6; age = 2.4; Age squared = 1.1.

### Modifiers of the relation between plasma NfL levels and retinal volume

**Figure 2:**
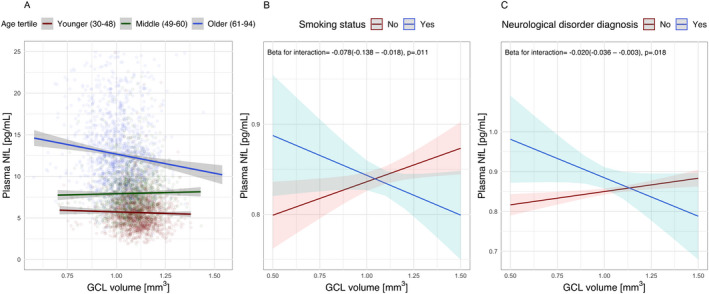
Modifiers of the relation between retinal measures and plasma NfL levels. The unadjusted association between GCL and plasma NfL largely depended on age (A). After accounting for all potential confounders, the relation between GCL volume and NfL levels was strongly dependent on the presence of (risk factors for) neurodegeneration, including smoking (B) and a history of neurological disorders (C). Abbreviations: SBP, systolic blood pressure; GCL, ganglion cell layer volume; NfL, neurofilament light chain. [Colour figure can be viewed at wileyonlinelibrary.com]

Effect modification was assessed for those retinal measures with the highest neuronal density (i.e., GCL and IPL) plus RPE, for which also a robust association with plasma NfL levels was observed (Table 1). The association between GCL and plasma NfL was strongest in the oldest age tertile (Figure 2A), but an interaction between GCL and age was not significant in the fully adjusted model. In active smokers and in participants with a history of neurological disorders, GCL thinning was associated with higher NfL levels (Table 1, Figure 2B,C). Results remained similar when utilizing IPL volume (Table 1).

The exclusion of individuals with intermediate age‐related macular degeneration (iAMD) did not change the statistical significance of any of our findings (data not shown).

## Discussion

We present the first large‐scale population‐based study assessing the comparative determinants and association of plasma NfL levels with OCT‐based retinal measures. Age, a history of neurological disorders and smoking largely accounted for the relationship between OCT‐based retinal layer measures and plasma NfL levels. This indicates that brain atrophy could underlie their association. Indeed, previous imaging studies found that age‐related brain atrophy starts in early adulthood, proceeds gradually throughout life and accelerates in old age. In line with these findings, we observed a similar age trend in both plasma NfL levels and GCL volume.^14^

Furthermore, we observed a non‐linear, U‐shaped, association between SBP and NfL levels, suggesting that both hypo‐ and hypertension may lead to neuronal injury,^15^ as well as a linear association between SBP and GCL. Hypertension is an established risk factor for small‐vessel disease and brain atrophy,^16^ further supporting the idea of brain atrophy underlying the association between plasma NfL and retinal neurodegeneration.

The RPE showed a positive association with NfL levels independent of age. This epithelial layer is a component of the blood–retinal barrier (BRB) and undergoes thickening, rather than thinning, during aging.^17^ Given the structural and functional similarities between the BRB and the blood–brain barrier (BBB),^18^ this finding might be explained by RPE volume reflecting BBB integrity rather than brain size.

The effects of GFR and blood volume on plasma NfL, reflecting its clearance and dilution rates, respectively, were also reported in two separate previous studies.^19^, ^20^ Our findings extend these findings by showing that their effects are mutually independent, which should be considered in future studies utilizing plasma NfL levels.

Correcting for age reversed the direction of association between GCL volume and NfL levels. To understand this phenomenon, one should distinguish between *brain atrophy* (brain volume change observed between two measurements) and *brain size* (cross‐sectional brain volume). We observed that in individuals with risk factors for brain atrophy, retinal neurodegeneration reflects rising plasma NfL levels. However, in individuals with the same rate of neuronal loss, once these risk factors are accounted for, a larger absolute brain size might lead to higher baseline NfL levels due to a larger amount of neuronal tissue available. Hence, both increasing brain atrophy and brain size may lead to higher NfL levels. Given that age is by far the strongest risk factor for neurodegeneration in the general population, adjusting for it largely removes the effect of brain atrophy, and amounts to comparing the association between NfL levels and GCL volume as a proxy for brain size.^3^, ^7^, ^8^ Supporting this notion are findings from a previous study showing a trend for a positive association between plasma NfL levels and brain volume in 99 individuals, although these findings need further confirmation in a larger population‐based cohort.^3^

The strengths of our study include a large population‐based sample, inclusion of measures of all retinal layers, and assessment and consideration of all known relevant confounders in our statistical analyses. Limitations include self‐reported history of medical conditions and lack of longitudinal data.

In conclusion, in the presence of risk factors for neuronal injury, plasma NfL levels are associated with inner retinal neurodegeneration and outer retinal thickening. Our study supports the integration of OCT‐based retinal measures as both clinical and research tools for tracking central neurodegeneration.

## Conflict of Interest

The author Robert P Finger declares grants and personal fees from Novartis; grants from Biogen; personal fees from Bayer, Santen, Ophtea, Apellis, Roche/Genentech, Böhringer‐Ingelheim, Novelion, ProQR, Oxford Innovation, Roche, Alimera, Santhera, Inositec, Ellex. Other authors declare no conflict of interest.

## References

[1] Khalil M , Teunissen C , Otto M , et al. Neurofilaments as biomarkers in neurological disorders. Nat Rev Neurol. 2018;14(10):577‐589. 30171200 10.1038/s41582-018-0058-z

[2] London A , Benhar I , Schwartz M . The retina as a window to the brain—from eye research to CNS disorders. Nat Rev Neurol. 2013;9:44‐53. 23165340 10.1038/nrneurol.2012.227

[3] Khalil M , Pirpamer L , Hofer E , et al. Serum neurofilament light levels in normal aging and their association with morphologic brain changes. Nat Commun. 2020;11(1). 10.1038/s41467-020-14612-6 PMC7010701 32041951

[4] Mauschitz MM , Holz FG , Finger RP , Breteler MMB . Determinants of macular layers and optic disc characteristics on SD‐OCT: the Rhineland study. Transl Vis Sci Technol. 2019;8(3):34. 10.1167/tvst.8.3.34 PMC6549562 31183250

[5] Fyfe I . Neurofilament light chain—new potential for prediction and prognosis. Nat Rev Neurol. 2019;15:557‐557. 31506590 10.1038/s41582-019-0265-2

[6] Mirzaei N , Shi H , Oviatt M , et al. Alzheimer’s retinopathy: seeing disease in the eyes. Front Neurosci. 2020;14. 10.3389/fnins.2020.00921 PMC7523471 33041751

[7] Saidha S , Al‐Louzi O , Ratchford JN , et al. Optical coherence tomography reflects brain atrophy in multiple sclerosis: a four‐year study. Ann Neurol. 2015;78(5):801‐813. 26190464 10.1002/ana.24487 PMC4703093

[8] Mutlu U , Bonnemaijer PWM , Ikram MA , et al. Retinal neurodegeneration and brain MRI markers: the Rotterdam study. Neurobiol Aging. 2017;60:183‐191. 10.1016/j.neurobiolaging.2017.09.003 28974335

[9] Tavazzi E , Jakimovski D , Kuhle J , et al. Serum neurofilament light chain and optical coherence tomography measures in MS. Neurol Neuroimmunol Neuroinflamm. 2020;7(4):e737. 32424064 10.1212/NXI.0000000000000737 PMC7251512

[10] Quanterix Corporation Simoa® NF‐light Advantage Kit. 1–3 (2018). Available at: https://www.quanterix.com/wp‐content/uploads/2020/12/NF‐light‐Data‐Sheet‐HD‐1∕HD‐X‐2.pdf

[11] Nadler SB , Hidalgo JH , Bloch T . Prediction of blood volume in normal human adults. Surgery. 1962;51:224‐232. 21936146

[12] Inker LA , Schmid CH , Tighiouart H , et al. Estimating glomerular filtration rate from serum creatinine and cystatin C. N Engl J Med. 2012;367(1):20‐29. 10.1056/nejmoa1114248 22762315 PMC4398023

[13] Benjamini Y , Hochberg Y . Controlling the false discovery rate: a practical and powerful approach to multiple testing on JSTOR. J R Stat Soc. 1995;57:289‐300.

[14] Peters R . Ageing and the brain. Postgrad Med J. 2006;82:84‐88. 16461469 10.1136/pgmj.2005.036665 PMC2596698

[15] Korte N , Nortley R , Attwell D . Cerebral blood flow decrease as an early pathological mechanism in Alzheimer's disease. Acta Neuropathol. 2020;140:793‐810. 32865691 10.1007/s00401-020-02215-w PMC7666276

[16] Zlokovic BV . Neurovascular pathways to neurodegeneration in Alzheimer's disease and other disorders. Nat Rev Neurosci. 2011;12:723‐738. 22048062 10.1038/nrn3114 PMC4036520

[17] Okubo A , Rosa, RH Jr , Bunce, CV , et al. The relationships of age changes in retinal pigment epithelium and Bruch's membrane. Investig Ophthalmol Vis Sci. 1999;40:443‐449. 9950604

[18] Steuer H , Jaworski A , Elger B , et al. Functional characterization and comparison of the outer blood–retina barrier and the blood–brain barrier. Investig Opthalmology Vis Sci. 2005;46(3):1047. 10.1167/iovs.04-0925 15728564

[19] Manouchehrinia A , Piehl F , Hillert J , et al. Confounding effect of blood volume and body mass index on blood neurofilament light chain levels. Ann Clin Transl Neurol. 2020;7(1):139‐143. 10.1002/acn3.50972 31893563 PMC6952306

[20] Akamine S , Marutani N , Kanayama D , et al. Renal function is associated with blood neurofilament light chain level in older adults. Sci Rep. 2020;10(1). 10.1038/s41598-020-76990-7 PMC7683708 33230211

